# Pheochromocytoma crisis combined with median arcuate ligament syndrome: A rare case report

**DOI:** 10.1097/MD.0000000000046794

**Published:** 2026-01-02

**Authors:** Jiang-xiong Wen, Wen-jia Li, Jing Guo, Hongnaly Siliath, Yun Xiao

**Affiliations:** a Department of Cardiovascular Internal Medicine, The First People’s Hospital of Yunnan Province, Affiliated Hospital of Kunming University of Science Technology, Kunming, Yunnan, China; b Department of Radiology, The First People’s Hospital of Yunnan Province, Affiliated Hospital of Kunming University of Science Technology, Kunming, Yunnan, China; c Department of Medical Intensive Care Unit, The First People’s Hospital of Yunnan Province, Affiliated Hospital of Kunming University of Science and Technology, Kunming, Yunnan, China; d Department of Cardiovascular Internal Medicine, Luang Prabang Provincial Hospital, Luang Prabang, Laos; e Department of Trauma Center, The First People’s Hospital of Yunnan Province, Affiliated Hospital of Kunming University of Science and Technology, Kunming, Yunnan, China.

**Keywords:** blood pressure fluctuations, catecholamine cardiomyopathy, median arcuate ligament syndrome, pheochromocytoma crisis

## Abstract

**Rationale:**

Left ventricular thrombus caused by acute catecholamine cardiomyopathy combined with pheochromocytoma crisis (PC) is extremely rare in clinical practice, and there are no reports of patients with median arcuate ligament syndrome (MALS) at the same time. This report describes the case of a 45-year-old woman admitted to the cardiology department with this rare condition. The patient presented with severe abdominal pain, vomiting, rapid blood pressure (BP) fluctuations, and recurrent hypotensive syncope. We discuss the patient’s diagnosis and treatment and share our experience with this patient.

**Patient concerns:**

A combination of PC and MALS has not been previously reported. This successful treatment provides a reference for the future treatment of similar patients, filling a related gap. The treatment methods and outcomes are the most important aspects of patient care.

**Diagnoses:**

The patient was admitted to the hospital because of chest tightness, headache, abdominal pain, and several episodes of syncope for 4 days. Clinical symptoms, combined with enhanced chest-abdominal computed tomography, 24-hour ambulatory BP monitoring, left and right coronary angiography, and renin-angiotensin-aldosterone and adrenocorticotropic hormone-cortisol measurements, led to a diagnosis of catecholamine cardiomyopathy, PC, and MALS.

**Interventions:**

Continuous invasive arterial and venous BP monitoring, norepinephrine was administered via an intravenous pump, and intravenous crystalloid infusions were administered to improve the patient’s significant BP fluctuations. Subsequently, after a gradual improvement in BP fluctuations, an alpha receptor blocker (phenoxybenzamine) was administered orally, and the dose was gradually increased to the target dose to control BP. Finally, after 6 months of optimized medical treatment, the right adrenal tumor was safely resected with complete recovery of cardiac structure and function.

**Outcomes:**

During the 6-month follow-up, after the operation, the patient’s BP was stable without medication, hypotensive syncope, recurrence of abdominal pain, and tumor recurrence. Key laboratory test results, such as cardiac Troponin I, N-terminal pro-B-type natriuretic peptide, white blood cell, adrenocorticotropic hormone, cortisol, etc, returned to normal levels

**Lessons:**

First, it highlights the effectiveness of norepinephrine in treating BP fluctuations and hypotensive syncope in patients with pheochromocytoma. Second, we propose a potential pathological interaction between pheochromocytoma and MALS, thereby avoiding surgical treatment of MALS. Third, it confirmed the safety of delayed surgery in patients with high-risk cardiac conditions.

## 1. Introduction

Pheochromocytoma (PHEO) produces catecholamines that can cause symptoms such as paroxysmal hypertension, palpitations, headaches, and sweating. Pheochromocytoma crisis (PC) is a rare manifestation of PHEO caused by abnormally high levels of catecholamine secretion, which can result in multi-organ damage. PC is defined as the presence of hypertension or hypotension, blood pressure (BP) fluctuations, catecholamine cardiomyopathy, encephalopathy, and other multi-organ damage.^[1]^ Hypertensive emergency due to PC complicated with refractory hemodynamic collapse. PC is associated with numerous symptoms and organs, making diagnosis and treatment challenging. Median arcuate ligament syndrome (MALS) is a rare disorder characterized by extrinsic compression of the celiac artery by the median arcuate ligament, resulting in nonspecific but typical clinical symptoms including postprandial abdominal pain, nausea, diarrhea, and weight loss.^[2,3]^ A combination of PC and MALS has not been previously reported. The unique pathological synergy between these 2 conditions aggravates PC severity. The successful treatment experience in this case provides a reference for the future treatment of similar patients.

## 2. Case Presentation

The study adhered to the tenets of the Declaration of Helsinki. The study was exempt from ethical approval by our institution. Written informed consent was obtained from the patient for publication of this case report and accompanying images.

**Figure 1.**
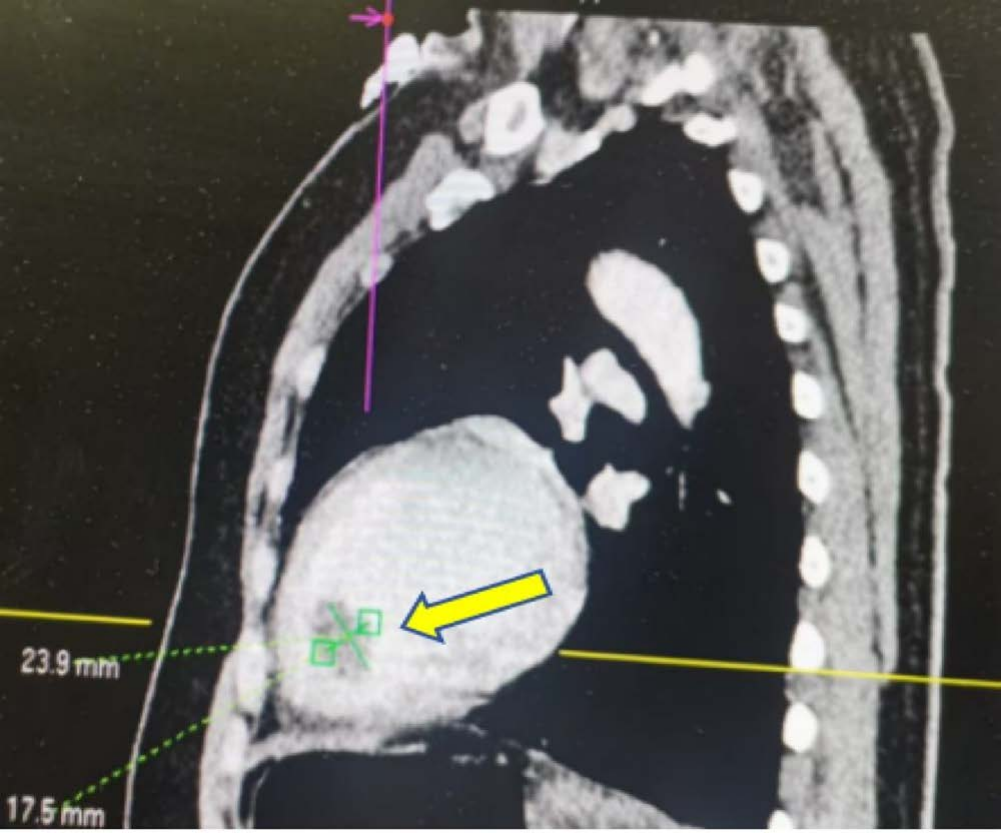
Contrast-enhanced CT scan showing an LV thrombus (yellow arrow). CT = computed tomography, LV = left ventricle.

**Figure 2.**
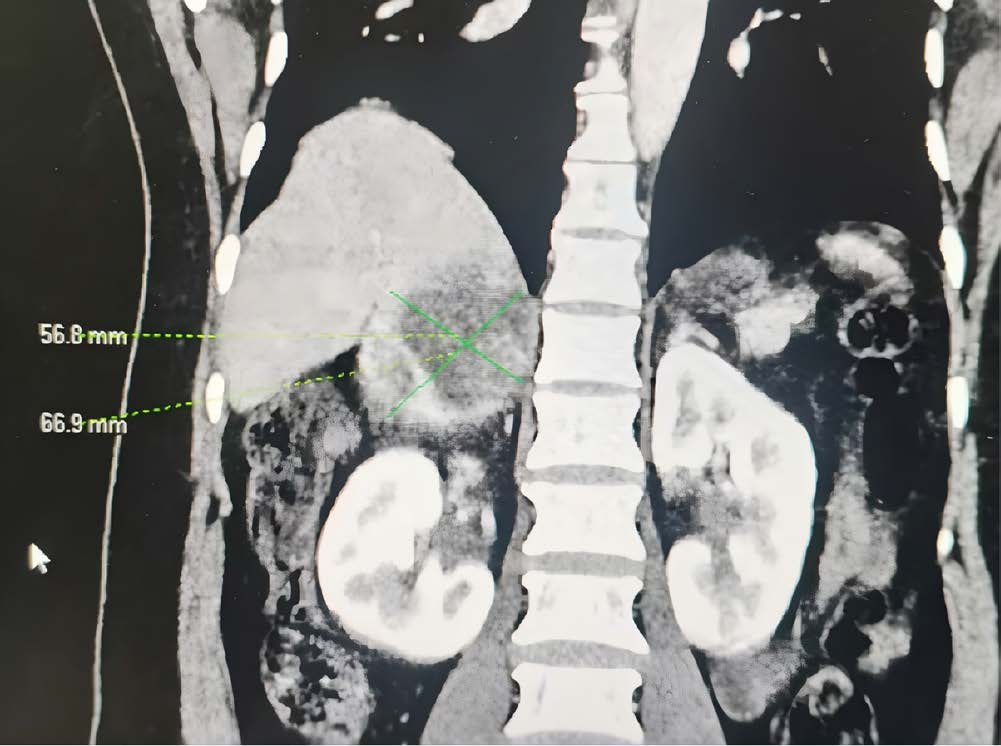
Contrast-enhanced CT scan showing a right adrenal mass (yellow arrow). CT = computed tomography.

**Figure 3.**
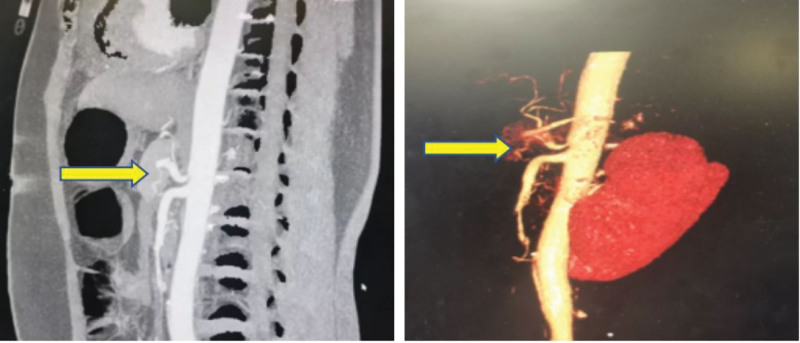
Contrast-enhanced CT: coronal reconstruction of celiac trunk compression (yellow arrow). CT = computed tomography.

**Figure 4.**
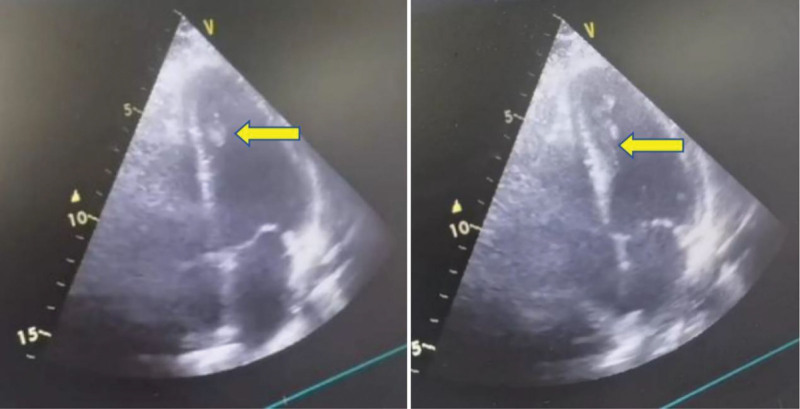
Bedside echocardiography revealing a left ventricular thrombus (yellow arrow).

A 45-year-old female was admitted to our hospital in September 2024, because of chest tightness, headache, and abdominal pain accompanied by multiple episodes of syncope for 4 days. She had a 6-year history of hypertension, irregular medication use, and unstable BP monitoring. She had no history of drug allergies, denied any history of diabetes, hepatitis, tuberculosis, or other infectious diseases, as well as alcohol consumption and smoking. Upon admission to our department, her temperature was 37.4°C, pulse rate ranged from 60 to 180 beats/min, respiratory rate ranged between 30 and 40 breaths/min, and BP ranged from 68/49 mm Hg to 190/120 mm Hg. The patient’s general condition was poor and she had a weak and painful appearance. She experienced a brief loss of consciousness that lasted several seconds, but then recovered on her own. The patient had a hot chest, cold extremities, no distended jugular vein, flushed face, profuse sweating, rapid breathing, coarse breath sounds in both lungs, few moist rales at the lung bases, enlarged cardiac silhouette, heart rate (HR): 60 to 180 beats/minute, regular rhythm, and no pathological murmurs. The key laboratory tests, along with their reference data and dynamic results, are listed in Tables 1–8. Imaging examination: enhanced chest-abdominal computed tomography (CT); suspected myocardial ischemia and possible ventricular thrombosis (Fig. 1), right adrenal mass (possibly PHEO; Fig. 2); severe stenosis at the origin of the celiac trunk (considered MALS; Fig. 3). Bedside echocardiography on admission revealed the following findings: multiple mural thrombi at the left ventricular apex (Fig. 4; maximum 1.0 cm × 2.2 cm, highly mobile); thickening of the basal ventricular septum and widening of the ascending aorta; decreased left ventricular systolic function (ejection fraction, 0.26) and grade II diastolic dysfunction. The initial diagnosis was hypotensive syncope, acute heart failure, left ventricular thrombus, catecholamine cardiomyopathy, right adrenal adenoma (PHEO), PC, MALS.

**Table 1:** Dynamic results of WBC test.

Date	WBC	Reference range	Unit
2024.09.05	16.88	3.50–9.50	10^9^/L
2024.09.10	17.15	3.50–9.50	10^9^/L
2024.09.20	8.89	3.50–9.50	10^9^/L
2024.09.28	7.61	3.50–9.50	10^9^/L
2024.12.31	5.27	3.50–9.50	10^9^/L
2025.03.05	3.83	3.50–9.50	10^9^/L
2025.03.10	4.85	3.50–9.50	10^9^/L
2025.03.13	9.56	3.50–9.50	10^9^/L
2025.03.19	10.97	3.50–9.50	10^9^/L

WBC = white blood cell.

**Table 2:** Dynamic results of HGB test.

Date	HGB	Reference range	Unit
2024.09.05	156	115–150	g/L
2024.09.10	112	115–150	g/L
2024.09.20	94	115–150	g/L
2024.09.28	95	115–150	g/L
2024.12.31	123	115–150	g/L
2025.03.05	142	115–150	g/L
2025.03.10	126	115–150	g/L
2025.03.13	128	115–150	g/L
2025.03.19	128	115–150	g/L

HGB = hemoglobin.

**Table 3:** Dynamic results of ALB test.

Date	ALB	Reference range	Unit
2024.09.05	37.8	40–55	g/L
2024.09.10	35.7	40–55	g/L
2024.09.20	30.6	40–55	g/L
2024.09.28	29.5	40–55	g/L
2024.12.31	34.9	40–55	g/L
2025.03.05	39.3	40–55	g/L
2025.03.13	36.6	40–55	g/L
2025.03.19	37	40–55	g/L

ALB = albumin.

**Table 4:** Dynamic results of NT-proBNP test.

Date	NT-ProBNP	Reference range	Unit
2024.09.05	1795	0–122.2	pg/mL
2024.12.04	412.5	0–122.2	pg/mL
2025.03.14	387.6	0–122.2	pg/mL
2025.03.15	489.6	0–122.2	pg/mL

NT-proBNP = N-terminal pro-B-type natriuretic peptide.

**Table 5:** Dynamic results of aTnI test.

Date	aTnI	Reference range	Unit
2024.09.05	3.253	0–0.0156	ng/mL
2024.09.20	0.080	0–0.0156	ng/mL
2024.12.31	0.020	0–0.0156	ng/mL
2025.01.07	0.020	0–0.0156	ng/mL
2025.03.05	0.010	0–0.0156	ng/mL
2025.03.14	0.005	0–0.0156	ng/mL
2025.03.15	0.004	0–0.0156	ng/mL

aTnI = cardiac troponin I.

**Table 6:** Dynamic results of DD2 test.

Date	DD2	Reference range	Unit
2024.09.05	5.46	0–0.5	µg/mL
2024.09.10	5.30	0–0.5	µg/mL
2024.09.20	5.02	0–0.5	µg/mL
2025.03.13	1.21	0–0.5	µg/mL
2025.03.19	1.57	0–0.5	µg/mL

DD2 = D-dimer.

**Table 7:** Dynamic results of COR8 test.

Date	COR8	Reference range	Unit
2024.09.05	13.14	4.82–19.50	µg/dL
2024.12.14	12.76	4.82–19.50	µg/dL
2025.03.05	12.56	4.82–19.50	µg/dL
2025.03.13	9.52	4.82–19.50	µg/dL
2025.03.20	5.64	4.82–19.50	µg/dL

COR8 = cortisol collected at 8 am.

**Table 8:** Dynamic results of ACTH8 test.

Date	ACTH8	Reference range	Unit
2024.09.05	26.35	7.2–63.3	pg/mL
2024.12.14	27.52	7.2–63.3	pg/mL
2025.03.05	44.45	7.2–63.3	pg/mL
2025.03.13	325.4	7.2–63.3	pg/mL
2025.03.20	14.23	7.2–63.3	pg/mL

ACTH8 = adrenocorticotropic hormone collected at 8 am.

The patient’s condition was extremely critical, with symptoms including significant chest tightness, abdominal pain, headache, and significant fluctuations in HR (60–180 beats/min) and BP (68/49–190/120 mm Hg), accompanied by hypotensive syncope. High-flow oxygen was administered via face mask to assist breathing, and 0.6 mg/h of norepinephrine (NE) was infused through a peripheral intravenous access to treat syncope caused by hypotension. The right internal jugular vein and right radial artery were punctured to maintain stable intravenous access and to monitor pressure fluctuations in real time. Additionally, symptomatic treatment, improvement of heart failure status, and the use of anticoagulants were administered for appropriate symptoms.

**Figure 5.**
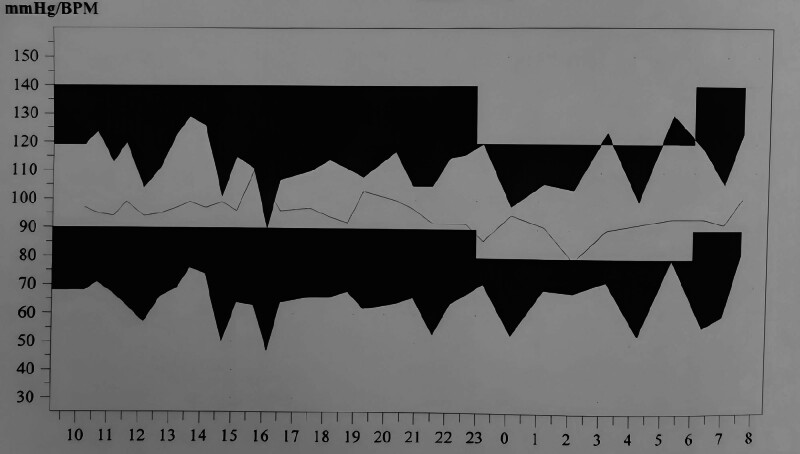
24-hour ambulatory BP. BP = blood pressure.

**Figure 6.**
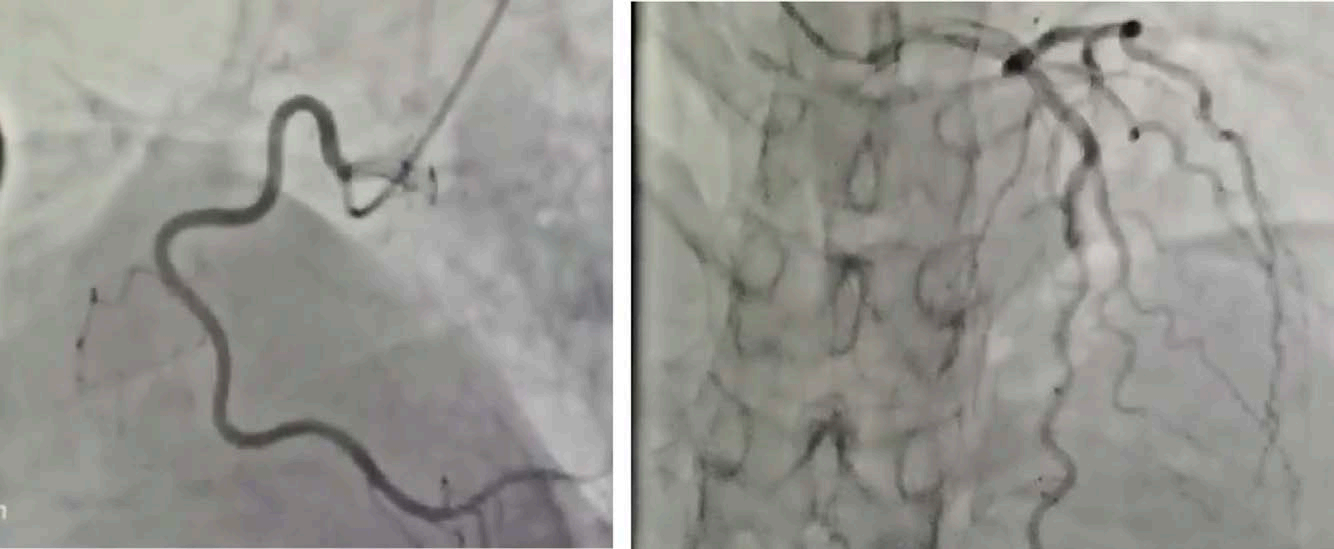
Left and right coronary angiography findings are normal.

The patient experienced abdominal pain, which worsened after eating, and vomiting, which was considered to be caused by MALS and excessive catecholamines. Therefore, the patient’s dietary intake was significantly reduced and daily physiological needs were supplemented intravenously. The medication and fluid infusion rates were adjusted in real time based on vital sign monitoring to maintain the relative hemodynamic stability. Contrast-enhanced CT of the chest and abdomen revealed a right adrenal mass. Further investigation of adrenal-derived secondary hypertension is warranted. Laboratory tests for renin-angiotensin-aldosterone and adrenocorticotropic hormone showed no abnormalities. All 6 blood catecholamine levels were significantly elevated, including a free NE level of 167,733.0 pmol/L (reference ranges: 414–4435 pmol/L) and a free epinephrine level exceeding 11,043.0 pmol/L (reference range: <605.4 pmol/L). After the patient was diagnosed with PHEO, oral phenoxybenzamine (phenoxybenzamine 10 mg/d, increased by 10–20 mg every 2–3 days until reaching 30–60 mg/d or 1 mg/(kg d)) was administered, in addition to intravenous low-dose NE (0.3 mg/h) and esmolol (60 mg/h). The patient’s fluctuating BP gradually stabilized (Fig. 5), and left ventricular ejection fraction recovered to 50%. On the sixth day of hospitalization, coronary angiography was performed. No stenosis was found in the right coronary artery, left main coronary artery, or circumflex branch (Fig. 6), ruling out a type I myocardial infarction and confirming acute catecholamine cardiomyopathy. Through meticulous management of intake and output, BP control, improvement of heart failure, and anticoagulation therapy, the patient’s chest tightness, abdominal pain, and other symptoms improved significantly, BP stabilized, cardiac function improved, and the volume of the left ventricular thrombus decreased. The patient’s general condition improved and he was discharged on the 15th day.

**Figure 7.**
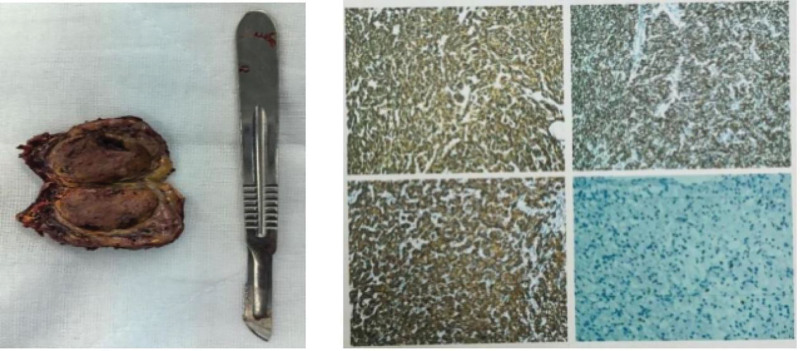
A tumor of the adrenal gland is excised. Hematoxylin and eosin–stained sections of resected adrenal tumors.

After discharge, the patient underwent regular outpatient follow-up and continued taking phenoxybenzamine, anticoagulants, and medication for chronic heart failure (including beta-blockers). Three months later, the left ventricular thrombus completely resolved, and the cardiac structure and function returned to normal (left ventricular ejection fraction, 64%). The patient was subsequently transferred to the urology department of our hospital for laparoscopic resection of a right adrenal tumor. During surgery, the right adrenal gland and the tumor were observed, measuring approximately 57 mm × 67 mm. Postoperative pathological immunohistochemistry results supported the diagnosis of PHEO (Fig. 7). The patient’s BP did not fluctuate significantly after the surgery and remained within the normal range. No antihypertensive medication was prescribed and the patient’s previous symptoms of abdominal pain and postprandial vomiting gradually became tolerable. The planned median arc ligament release surgery was canceled in favor of conservative treatment. Over the next 3 months, during regular outpatient follow-up, her BP returned to normal and she no longer required medication. The patient experienced no chest distress, pain, or abdominal pain after meals. She was able to perform modest household chores. The patient was very satisfied with the medical and surgical treatment that she received.

## 3. Discussions

PHEOs are rare. It is an abnormal proliferation of chromaffin cells in adrenal glands. These cells secrete large amounts of catecholamines that can cause hypertension. Even rare cases of PHEO can cause multiple organ damage.^[1]^ Some patients experienced significant fluctuations in BP. High BP can cause headaches and worsen heart failure, whereas low BP may lead to syncope.^[4–6]^ However, the mechanisms underlying hypotension and BP fluctuations in these patients remain unclear. Decreased blood volume due to inhibition of the renin-angiotensin-aldosterone system and downregulation of adrenergic receptors contributes to the hypotension observed in patients with PHEO.^[7]^ Furthermore, there is no consensus on the management of hypotension and BP fluctuations caused by PHEO. Consequently, these patients face a dilemma when choosing treatment options. According to previous reports by Fujii et al, intravenous NE can help improve BP fluctuations, particularly hypotension. Therefore, for the management of BP fluctuations and severe hypotension with syncope, we chose intravenous NE rather than phentolamine, which may worsen hypotension.^[8]^

The clinical symptoms of MALS are caused by the compression of the celiac artery by the median arcuate ligament, leading to ischemia of the corresponding gastrointestinal tissues, including postprandial abdominal pain and nausea. If patients exhibit significant ischemic symptoms, most patients require surgical intervention.^[2,3]^ Anatomically, this is associated with the morphology of the celiac artery, specifically, the origin of the celiac artery trunk from the abdominal aorta being too high or the attachment point of the diaphragmatic crura being too low, both of which can lead to compression of the celiac artery. Additional imaging studies, such as CT or magnetic resonance angiography, can help identify the extent of mesenteric stenosis and rule out other potential organic diseases.^[2,9]^ MALS can lead to a significant reduction in food intake, which can cause a hypovolemic state and further complicate the condition of PHEO patients. Our patient had severe abdominal pain and vomiting, for which the gastrointestinal surgeon recommended surgical treatment. However, considering that the patient’s abdominal pain might have been caused by the release of catecholamines from the PHEO, we did not adopt this suggestion and chose conservative treatment instead. Fortunately, after the adrenal tumor was removed and conservative treatment was administered, the patient’s abdominal pain and vomiting disappeared.

There have been no previous reports on the co-occurrence of PHEO and MALS, suggesting that these 2 disorders may interact. NE, secreted by PHEO, acts on the α1 receptors of the distal small vessels of the celiac artery, causing vasoconstriction and spasms. Simultaneously, mechanical compression of the celiac artery caused by MALS exacerbates gastric/intestinal mesenteric ischemia, leading to more severe postprandial abdominal pain and vomiting. Hypovolemia and sympathetic nervous system activation further exacerbate BP fluctuations and catecholamine release.^[10,11]^ A potential “double hit” mechanism (first hit: the catecholamines secreted by PHEO aggravate the celiac ischemia caused by MALS; second hit: postprandial abdominal pain and vomiting due to MALS exacerbate the BP fluctuations caused by PHEO) may worsen the condition of the pathient. After noticing the potential “double hit,” we provided the corresponding treatment measures. For abdominal pain and vomiting caused by MALS, which made the patient reluctant to eat, we provided parenteral nutrition to supplement the necessary energy and fluids based on real-time monitoring of vital signs, such as BP and central venous pressure, to maintain effective circulating blood volume. Simultaneously, we administered epinephrine via an intravenous pump and gradually adjusted the dose of oral phenoxybenzamine to reduce BP fluctuations and sensitivity to catecholamines. Fortunately, through the above measure, the patient’s significant BP fluctuations and hypoglycemic syncope significantly improved. As BP control improved, the postprandial abdominal pain gradually improved. This confirmed our hypothesis that the combined effects of PC and MALS resulted in insufficient blood volume, which caused significant fluctuations in the patient’s BP and triggered syncope. It is important to note that the exact pathophysiological mechanism underlying the combined effects of PC and MALS on the body remains unclear and requires further investigation.

Clinical practice guidelines recommend surgical treatment of PHEO. However, there is no clear time frame for surgery.^[12]^ PHEO resection is a high-risk surgery, and thorough preoperative preparation is crucial. The full use of α- and β-adrenergic receptor blockers is recommended. It has been reported that α- and β-adrenergic receptor blockers can inhibit sympathetic nerve activity, thus reducing surgical mortality.^[13]^ Given that the patient’s cardiac structure and function suffered severe damage, we did not rush to arrange the surgery for the patient. Instead, we arranged for regular outpatient follow-up and prescribed medications outside the hospital, including phenoxybenzamine, anticoagulants, and myocardial nutrition drugs. We used pulsed wave tissue Doppler imaging to regularly monitor the motion of the patient’s cardiac structure and assess the risk of left ventricular thromboembolism.^[14]^ Sugery is performed after the cardiac structure and function are fully restored and the left ventricular thrombus is completely eliminated. The urologist successfully resected the right adrenal tumor with an optimal dosage of α- and β-receptor blockers.

## 4. Conclusions

PC leads to acute catecholaminergic cardiomyopathy and significant BP fluctuations. Some patients experience recurrent syncope due to hypotension, which can be life threatening. PC and MALS synergistically exacerbate BP fluctuations and gastrointestinal ischemic symptoms. Therefore, both conditions must be addressed simultaneously during treatment. Based on adequate replenishment of the effective circulating blood volume, NE can effectively reduce significant fluctuations in BP and prevent the occurrence of hypotensive syncope. After adrenalectomy, MALS-related symptoms resolved, highlighting the need for surgical intervention. During follow-up within 3 months after surgery, the patient’s BP, HR, and plasma catecholamine levels remained normal without the need for medical therapy. The potential pathological association between PHEO and MALS remains unclear and requires further research. More research is needed to determine the optimal surgical time for PHEO resection.

## Author contributions

**Conceptualization:** Yun Xiao, Jiang-xiong Wen.

**Data curation:** Wen-jia Li.

**Supervision:** Jing Guo.

**Validation:** Wen-jia Li.

**Visualization:** Jing Guo.

**Writing – original draft:** Jiang-xiong Wen.

**Writing – review & editing:** Yun Xiao, Hongnaly Siliath.
